# Altered Epithelial Gene Expression in Peripheral Airways of Severe Asthma

**DOI:** 10.1371/journal.pone.0168680

**Published:** 2017-01-03

**Authors:** Akul Singhania, Hitasha Rupani, Nivenka Jayasekera, Simon Lumb, Paul Hales, Neil Gozzard, Donna E. Davies, Christopher H. Woelk, Peter H. Howarth

**Affiliations:** 1 Clinical and Experimental Sciences, Faculty of Medicine, University of Southampton, Southampton, United Kingdom; 2 UCB Celltech, Slough, United Kingdom; 3 Southampton NIHR Respiratory Biomedical Research Unit, Southampton Centre for Biomedical Research, University Hospital Southampton NHS Foundation Trust, Southampton, United Kingdom; Cincinnati Children's Hospital Medical Center, UNITED STATES

## Abstract

Management of severe asthma remains a challenge despite treatment with glucocorticosteroid therapy. The majority of studies investigating disease mechanisms in treatment-resistant severe asthma have previously focused on the large central airways, with very few utilizing transcriptomic approaches. The small peripheral airways, which comprise the majority of the airway surface area, remain an unexplored area in severe asthma and were targeted for global epithelial gene expression profiling in this study. Differences between central and peripheral airways were evaluated using transcriptomic analysis (Affymetrix HG U133 plus 2.0 GeneChips) of epithelial brushings obtained from severe asthma patients (N = 17) and healthy volunteers (N = 23). Results were validated in an independent cohort (N = 10) by real-time quantitative PCR. The IL-13 disease signature that is associated with an asthmatic phenotype was upregulated in severe asthmatics compared to healthy controls but was predominantly evident within the peripheral airways, as were genes related to mast cell presence. The gene expression response associated with glucocorticosteroid therapy (i.e. *FKBP5*) was also upregulated in severe asthmatics compared to healthy controls but, in contrast, was more pronounced in central airways. Moreover, an altered epithelial repair response (e.g. *FGFBP1*) was evident across both airway sites reflecting a significant aspect of disease in severe asthma unadressed by current therapies. A transcriptomic approach to understand epithelial activation in severe asthma has thus highlighted the need for better-targeted therapy to the peripheral airways in severe asthma, where the IL-13 disease signature persists despite treatment with currently available therapy.

## Introduction

Severe asthma represents a spectrum of the disease that is inadequately responsive to therapy and remains difficult to control [1]. Glucocorticosteroids, which comprise the currently available asthma therapy, reduce airway inflammation and directly target many inflammatory and structural cells involved in inflammation such as epithelial cells [2]. Bronchial epithelial cells are central to the host tissue response to factors in the external environment, being responsive and orchestral, in signalling to the tissue mesenchymal cells [3] and host adaptive immune response [4]. Thus studies undertaking bronchial brushings to recover bronchial epithelial cells have been highly informative in describing changes in gene expression in asthma [5, 6]. This has linked IL-13 response genes, *chloride channel*, *calcium-activated*, *family member 1 (CLCA1)*, *periostin (POSTN)*, *and serine peptidase inhibitor*, *clade B*, *member 2 (SERPINB2)* to a Th2 asthmatic endotype and a measure for steroid responsiveness [5, 6]. These studies have, however, restricted assessments to large central airways and have predominantly focused on milder asthma. It is now well established that asthma is an umbrella term with distinct diseases [7]. As such, there is limited information about severe asthma, which remains refractory to the current glucocorticosteroid therapy, and no information about epithelial cell activation in peripheral airways of such patients.

Peripheral airways account for majority of the luminal surface area within the airways, representing 98% of the airway surface area [8]. They are increasingly appreciated to be of significance to clinical disease expression in asthma [9–11]. Although direct evaluation of airway tissue changes at this site is difficult, transbronchial biopsies [12–14] and surgical specimens [15] in addition to post-mortem studies of asthmatic patients dying from asthma, as well as from non-asthmatic causes, have identified both inflammatory and structural airway changes in peripheral airways. Moreover, previous studies have demonstrated that peripheral airways are capable of producing Th2 cytokines [16, 17]. This highlights the need for targeted assessments in these smaller peripheral airways including the use of transcriptomic approaches.

To investigate the contribution of peripheral airways to disease persistence in severe asthma, epithelial cells were collected using fibre-optic bronchoscopy from central airways and peripheral airways. Owing to the heterogeneity in asthma, this study focused on the severest form of disease that has the greatest unmet need. Treatment-resistant severe asthma patients were recruited for comparison to healthy non-asthmatic controls, in order to understand what differences existed from normality. Samples were subjected to microarray analysis and results were validated in a separate independent cohort by real-time quantitative PCR (RT-qPCR).

## Materials and Methods

### Study population

Seventeen severe asthma patients with inadequate disease control, despite treatment at GINA management stage 4 or 5, and 23 non-asthmatic healthy volunteers were recruited (Table 1), with paired airway samples from majority of the subjects (Table 2). The severe asthmatics were all receiving high dose inhaled steroid therapy plus additional controller therapy (all LABA plus in many additional maintenance therapy [LTRA and theophylline preparations]), as well as in those at step 5 additional daily maintenance oral steroid therapy. All severe asthmatics had a history of at least 2 exacerbations in the last year and 44% had a history of hospital admission for acute severe asthma in the last year. All subjects were current non-smokers while 3 were ex-smokers with a pack year history of <2 and had ceased smoking for at least 1 year. An independent clinically similar cohort of 5 severe asthmatics and 5 healthy volunteers was also recruited for validation by RT-qPCR (Table 1). The study was approved by the Southampton and South West Hampshire Local Research Ethics Committee and all subjects gave written informed consent.

**Table 1:** Clinical characteristics of subjects who participated in the microarray and RT-qPCR study.

Clinical variable	MICROARRAY			RT-qPCR		
	Health	Severe asthma	*P* value	Health	Severe asthma	*P* value
	(n = 23)	(n = 17)		(n = 5)	(n = 5)	
Age (years), mean (range)	26 (19–54)	41 (20–63)	**<0.001**	35 (34–67)	55 (35–75)	0.15
Gender (Male), n (%)	14 (60.87)	9 (52.94)	0.859	3 (60)	2 (40)	0.71
Allergic SPT (Positive), n (%)	3 (13.64)	13 (76.47)	**<0.001**	-	3 (60)	NA
BTS asthma scale, n (%) 4 5	NA	12 (70.59) 5 (29.41)	NA	NA	2 (40) 3 (60)	NA
% predicted FEV_1,_ mean (range)	103.1 (83–141)	64.3 (26.5–123)	**<0.001**	110.3 (100–120)	67.7 (56.6–96)	**0.002**
Reversibility (% FEV_1_), mean (range)	4.7 (0–12)	18.2 (0–54)	**0.021**	5.7 (3–9.6)	20.7 (10–29)	**0.02**
ICS dose (μg/d), mean (range)	NA	2729 (2000–4400)	NA	NA	3300 (2400–5200)	NA
OCS (yes), n (%)	NA	4 (23.53)	NA	NA	3 (60)	NA
FeNO (ppb), mean (range)	11.47 (1–31)	15.81 (2–33.65)	0.263	21.5 (19–25.5)	38.75 (20.5–57)	0.4
BAL (%), mean (range) Macrophages Neutrophils Epithelial cells Eosinophils Lymphocytes	84.1 (53–97.3) 3.2 (0.3–42.5) 10.2 (0.3–42.5) 0.4 (0–1.5) 1.9 (0–5.3)	61.4 (7.3–91) 10.7 (1.3–68) 24.1 (3.5–89.8) 2.9(0–30.3) 1.0 (0–3)	**0.003** **0.028** **0.020** 0.619 0.056	92.4 (78–98) 0.9 (0.3–1.5) 6.0 (0.4–20) 0.2 (0–0.6) 0.03 (0–0.1)	63.5 (33–85.4) 15.6 (2.1–30.6) 13.2 (0.4–33.9) 2.2(0–9) 0.05 (0–0.3)	**0.029** **0.016** 0.556 0.522 1

Continuous variables were tested by Student *t* test if parametric, or by Wilcoxon test if non-parametric. Categorical variables were analysed using proportions test. *SPT*, skin prick testing; *BTS*, British Thoracic Society; *FEV1*, forced expiratory volume in 1 second; *ICS*, inhaled corticosteroids; *μg/d*, microgram per day; *OCS*, oral corticosteroids; *FeNO*, fractional exhaled nitric oxide; *ppb*, parts per billion; *BAL*, bronchoalveolar lavage; *pg/m*l, picogram per milliliter; *NA*, not applicable.

**Table 2:** Sample numbers for subjects who participated in the microarray study.

SEVERE ASTHMA (n = 17)			HEALTH (n = 23)		
Patient number	Central airways	Peripheral airways	Volunteer number	Central airways	Peripheral airways
1	P	P	1	P	P
2	P	P	2	P	P
3	P	P	3	P	P
4	P	P	4	P	P
5	P	P	5	P	P
6	P	P	6	P	P
7	P	P	7	P	P
8	P	P	8	P	P
9	P	P	9	P	P
10	P^§^	P^§^	10	P	P
11	P^§^	P^§^	11	P	P
12	P^§^	A^†^	12	P	P
13	P	A^#^	13	P	P
14	A^†^	P^§^	14	P	P
15	A^#^	P	15	P	P
16	A^#^	P	16	P	P
17	A^#^	P	17	P	P
-	-	-	18	P	P
-	-	-	19	P	P
-	-	-	20	P	A^#^
-	-	-	21	P	A^#^
-	-	-	22	P	A^#^
-	-	-	23	P	A^#^
Total (n)	13	15	Total (n)	23	19

*P*, sample present; *A*, sample absent;

^§^, patients on oral corticosteroids;

^†^, sample not collected;

^#^, sample removed due to poor RNA quality.

### Sample collection

Epithelial brushings were collected using flexible optic bronchoscopy under local anaesthesia, as per established guidelines. Four separate sets of brushings from central airways and four from peripheral airways were collected using disposable, sheathed bronchial brushes (Olympus BC-202D-1210). Central brushes were taken under direct vision from the right bronchus intermedius. Peripheral brushings were obtained by extending the sheathed brush (external diameter 1.2 mm) out of vision as far as possible into a right lower lobe sub-segmental bronchus, until an appreciation of localised awareness was obtained by the volunteer suggestive of a pleural response. This was followed by slight retraction and extension of the brush (diameter 0.064 mm) beyond the sheath. RNA was extracted using the Qiagen miRNeasy Kit (Qiagen Ltd., UK) and quality assessed using a Bioanalyzer 2100 (Agilent Ltd., UK). Samples were hybridized to Affymetrix HG U133 plus 2.0 GeneChips. Gene expression data are available at Gene Expression Omnibus (http://www.ncbi.nlm.nih.gov/geo) under accession number **GSE64913**.

### Microarray analysis

Raw microarray gene expression data was normalized using GeneChip Robust Multi-Array Analysis (GCRMA) [18] and subjected to quality control procedures as previously described [19]. Principal component analysis [20] revealed two distinct batches based on the year of microarray hybridization. These batch effects were removed using *sva* (surrogate variable analysis) [21] in R as the number of central and peripheral airway samples from health as well as severe asthma were included in both batches (S1 Fig). The smoking status of subjects did not have an effect on the gene expression (S2 Fig) and all subjects were retained for further analysis. A PCA plot was also generated to evaluate the effect of disease status and airway type (S3 Fig). Differentially expressed genes were identified using *limma* [22] in R. Gene ontology analysis was performed using ToppGene [23] and the resulting terms were collapsed into categories of related terms using REVIGO [24]. A semantic similarity-integrated approach for modularization (SSIM) [25] was also applied to the differentially expressed genes to find modules of functionally related genes based on co-expression, protein-protein interaction and shared gene ontology.

### RT-qPCR analysis

RNA was isolated and reverse transcribed into cDNA using the High Capacity cDNA Reverse Transcription Kit (Life Technologies). RT-qPCR was performed as previously described [26] for 6 genes in duplicate using Taqman Universal PCR Master Mix, No AmpErase UNG on 7900HT Fast Real-Time PCR System (Life Technologies). Changes in gene expression were calculated using the relative quantification (RQ) method (2^-ΔΔCT^) with *GAPDH* as a normalizer.

### Statistical analysis

In microarray analysis, a linear mixed modelling approach was used for differential gene expression analysis taking pairing of samples into account. Age mismatch between severe asthmatics and healthy volunteers (Table 1) was removed by fitting age in the linear model. Atopy mismatch between severe asthma and health (Table 1) could not be removed using the linear model due to very few healthy volunteers being atopic, however, comparisons between atopic and non-atopic subjects within peripheral and central airways did not generate any differentially expressed genes (data not shown). Only genes with False Discovery Rate (FDR) p-values < 0.05 corrected for multiple testing using the Benjamini-Hochberg (BH) method [27] were considered significant. A targeted interaction analysis was also performed by setting up contrasts in a linear mixed modelling approach [28] to evaluate the effects of disease and lung airway region on gene expression. In RT-qPCR analysis, a Mann-Whitney test was performed in a pairwise manner and p-values < 0.05 were considered significant. All groups in the RT-qPCR data are displayed on the same individual graph for visual purposes only.

## Results

### Peripheral airways versus central airways

**Fig 1:**
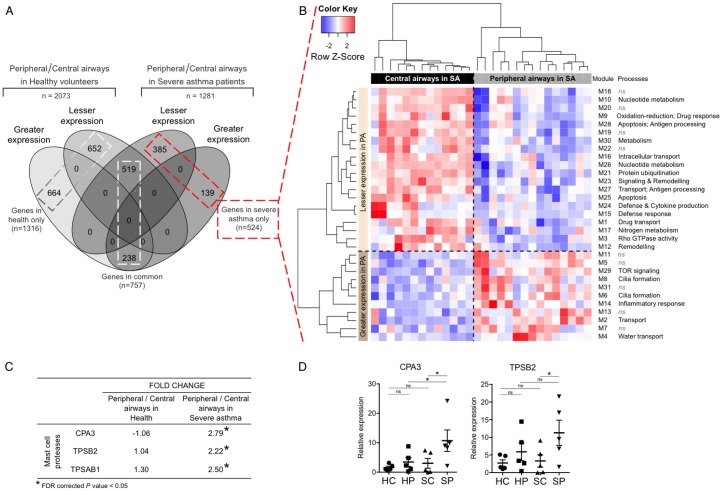
Peripheral airways versus central airways, in healthy volunteers and in severe asthmatics. **A.** *Greater expression*, genes with a greater expression in peripheral airways compared to central airways; *Lesser expression*, genes with a lesser expression in peripheral airways compared to central airways. **B.** Heatmap depicting unsupervised hierarchical clustering (Pearson complete) of modules identified using SSIM. Expression values of genes within each module were averaged and scaled to indicate the number of standard deviations above (red) or below (blue) the mean, denoted as row Z-score. *PA*, peripheral airways; *SA*, severe asthmatics; *ns*, not significant. **C.** Fold changes for mast cell proteases obtained by differential gene expression analysis. **D.** RT-qPCR validation of differential gene expression analysis of mast cell proteases. Changes in gene expression (mean and standard error of mean) are shown relative to *GAPDH*. *HC*, central airways in health; *HP*, peripheral airways in health; *SC*, central airways in severe asthma; *SP*, peripheral airways in severe asthma; *, p-value < 0.05.

In health, 2,073 genes were identified as differentially expressed between peripheral airways and central airways, indicating a large biological distinction between the two airways sites. In severe asthma, 1,281 genes were differentially expressed between the airways (Fig 1A, S1 Table; a positive fold change indicates higher expression of the gene in peripheral compared to central airways and a negative fold change indicates lower expression in peripheral compared to central airways). A core set of 757 genes was consistently differentially expressed between the airway sites in health as well as in severe asthma (Fig 1A) with the same direction of fold changes, suggesting their importance in maintaining the inherent biology of the airways even in the disease state. Gene ontology analysis for these 757 genes identified three representative categories: *translational termination*, *intrinsic apoptotic signaling pathway in response to DNA damage* and *anatomical structure formation involved in morphogenesis* (data not shown).

Of the 1281 genes differentially expressed between the airways in severe asthma, a set of 524 genes was identified as differentially expressed only in severe asthma and not in health (Fig 1A). These genes were subjected to an SSIM analysis, which integrates gene-gene expression correlations, curated protein-protein interactions and gene ontology annotations. This approach identified 31 gene modules that were enriched for biological processes (Fig 1B, S2 Table). Eleven modules consisted of genes with greater expression in peripheral airways compared to central airways and represent processes more active in peripheral airways in severe asthma. These included cilia formation (M6, M8), transport (M2, M4) and inflammatory response (M14). Module M31 included the IL-13 response gene, *CLCA1* and the mast cell marker *carboxypeptidase A3* (*CPA3*), suggesting a greater expression of mast cell secreted proteases in peripheral airways compared to central airways in severe asthma. This was further supported by the upregulation of mast cell tryptases: *tryptase β2* (*TPSB2*) and *tryptase α/β1* (*TPSAB1*) (Fig 1C) in module M14. Differential expression of *CPA3* and *TPSB2* between central and peripheral airways in severe asthma was confirmed by RT-qPCR (Fig 1D).

Conversely, the other 20 modules identified using SSIM consisted of genes with lesser expression in peripheral airways compared to central airways representing processes that may be expressed to a greater degree in central airways (Fig 1B). These included drug transport (M1, M9), apoptosis and remodelling (M3, M12, M25), signal transduction (M23), metabolism (M10, M17, M26, M30), and defence response (M15, M24, M27, M28). Module M1 was enriched for carboxylic acid transport, a process relevant to drug transport, suggestive of a greater glucocorticosteroid response in the central airways.

### Severe asthma versus health

**Fig 2:**
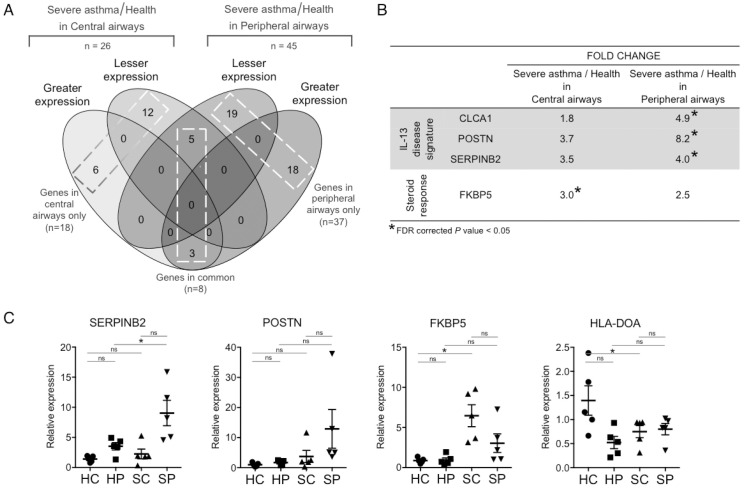
Severe asthma versus health, in central airways and in peripheral airways. **A.** *Greater expression*, genes with a greater expression in severe asthmatics when compared to healthy volunteers; *Lesser expression*, genes with a lesser expression in severe asthmatics when compared to healthy controls. **B.** Fold changes for IL-13 disease signature and steroid response obtained by differential gene expression analysis. **C.** RT-qPCR validation of microarray findings. Changes in gene expression (mean and standard error of mean) are shown relative to *GAPDH*. *HC*, central airways in health; *HP*, peripheral airways in health; *SC*, central airways in severe asthma; *SP*, peripheral airways in severe asthma; *, p-value < 0.05.

A cross-sectional comparison between severe asthma and health identified 37 genes differentially expressed in peripheral airways only, 18 genes differentially expressed in central airways only and 8 genes consistently differentially expressed across both airways (Fig 2A, S3 Table; a positive fold change indicates higher expression of the gene in severe asthma compared to health and a negative fold change indicates lower expression in severe asthma). In peripheral airways, these 37 differentially expressed genes included the greater expression of the IL-13 response gene signature (*CLCA1*, *POSTN*, *SERPINB2*) in severe asthmatics compared to healthy controls (Fig 2B), as well as *proline rich 4* (*PRR4*) and *mucin 12* (*MUC12*). FK506 binding protein 5 (*FKBP5*), a common glucocorticosteroid response marker, however, did not attain significance across peripheral airways (Fig 2B). The significant upregulation of *SERPINB2* in peripheral airways of severe asthmatics compared to healthy controls was confirmed by RT-qPCR, as was the direction of fold change of *POSTN* (Fig 2C).

Prominent amongst the 18 genes significantly differentially expressed within the central airways was the glucocorticosteroid response marker *FKBP5*, which had greater expression in severe asthma compared to health (Fig 2B, S3 Table). The IL-13 response signature (*CLCA1*, *POSTN*, *SERPINB2*) was also upregulated in the central airways in severe asthma compared to health, however, to a lesser extent compared to peripheral airways, and it did not attain significance (Fig 2B). Moreover, *Chemokine (C-C Motif) Ligand 5* (*CCL5*) was significantly reduced within the central airways in severe asthma, as was the epithelial gene expression for the *MHC class II*, *DO alpha* (*HLA-DOA*), which regulates peptide loading and is involved in antigen presentation. The significant upregulation of *FKBP5* and downregulation of *HLA-DOA* in the central airways of severe asthmatics compared to healthy controls was confirmed by RT-qPCR (Fig 2C).

The 8 genes consistently differentially expressed in severe asthma compared to health across both airways exhibited consistent fold changes and included genes involved in cytoskeletal structure regulation, cell proliferation and differentiation, such as *keratin 23* (*KRT23*), *fibroblast growth factor binding protein 1* (*FGFBP1*) and a chemokine receptor type 7 (*CXCR7*) (S3 Table). These findings are consistent with an ongoing epithelial repair response throughout the airways.

### Interaction analysis

**Fig 3:**
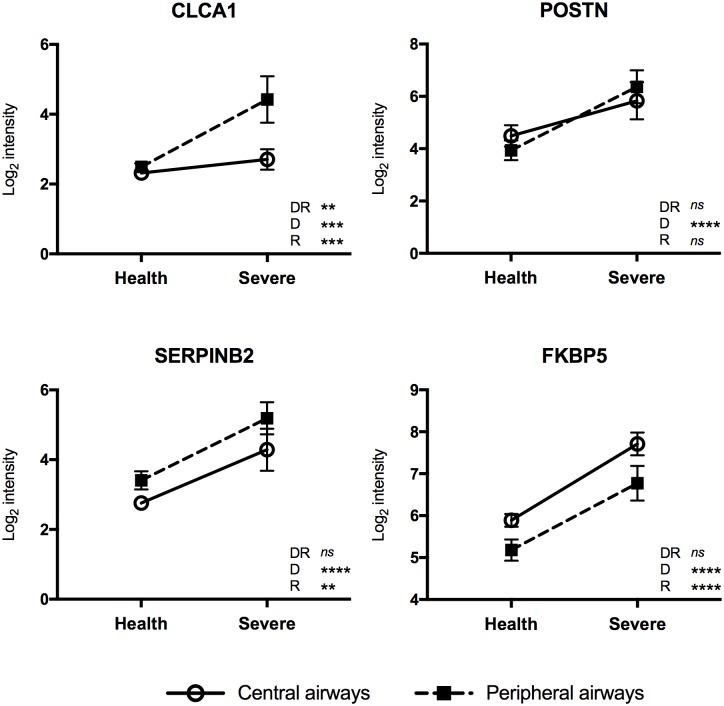
Interaction analysis to evaluate the effects of disease and lung airway region on gene expression. Log_2_ intensities are plotted and significance of interaction is depicted by: *DR*, interaction between disease and lung airway region; *D*, main effect of disease; *R*, main effect of region; p-values * < 0.05, ** < 0.01, *** <0.001, **** <0.0001; *ns*, not significant.

An interaction analysis was performed to evaluate the effects of disease and lung airway region on gene expression for genes constituting the IL-13 signature and *FKBP5*. Significant effects for both disease and lung region were seen for *CLCA1*, *SERPINB2* and *FKBP5* (Fig 3). In the case of *CLCA1* a non-crossing interaction was also significant. *CLCA1* and *SERPINB2* were upregulated in severe asthma and also in peripheral airways suggesting the persistence of the IL-13 signature in this lung region. *FKBP5* was also upregulated in severe asthma indicating exposure to steroids but was expressed to a lower extent in peripheral airways suggesting that treatment is not exerting the same biological effect in this lung region. Finally, there is only a significant effect for disease for *POSTN*, which was upregulated in severe asthma, but did not appear to be affected by lung region.

### Heterogeneity in the severe asthma samples

**Fig 4:**
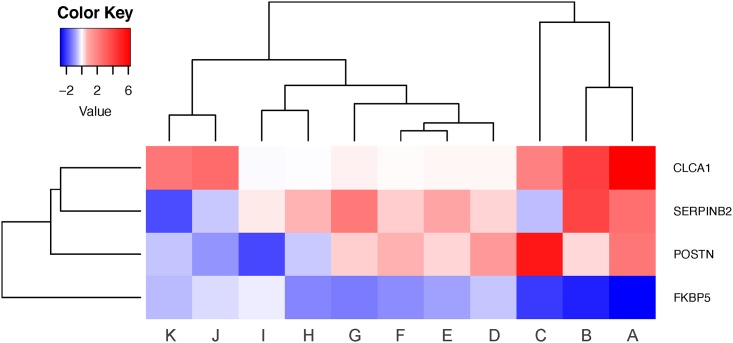
Heatmap depicting heterogeneity in severe asthma samples. Unsupervised hierarchical clustering (Euclidean complete) was performed on log_2_ fold change values for severe asthmatics with paired samples from both central and peripheral airways (N = 11 pairs). Red and blue represent greater and lower expression in the peripheral airways compared to the central airways, respectively. Severe asthma patients have been assigned a letter from A through K, arbitrarily.

Heterogeneity in the severe asthma samples was evaluated using the IL-13 disease signature (*CLCA1*, *SERPINB2* and *POSTN*) and the gene expression marker for steroid response (*FKBP5*). Severe asthma patients with paired samples from both central and peripheral airways were evaluated (N = 11 pairs, Table 2) using a fold change heatmap, such that red represents higher expression and blue represents lower expression in the peripheral airways compared to the central airways. Heterogeneity was evident in these severe asthmatics as subsets of patients formed distinct clusters (Fig 4). Three patients on the right (patients A through C) displayed a strong IL-13 disease signature persisting in their peripheral airways and a poor response to therapy. The next major cluster consisting of a subset of six patients (patients D through I) followed a similar pattern albeit to a lesser extent. In the remaining two patients (patients J and K), although the peripheral airways steroid response was still worse compared to the central airways, it was effective in reversing some of the disease signature. A strong response to *CLCA1*, however, still persisted in these patients. These results indicate that there is heterogeneity in these severe asthma patients. However, a lesser and inadequate response to steroid therapy is evident in the peripheral airways across all patients compared to their central airways as demonstrated by the reduced expression of FKBP5 in the peripheral airways. These findings highlight the heterogeneity of the biology in severe asthma and the importance of taking this into account in intervention studies as well as the relevance of site of airways disease (central versus peripheral airways).

### Oral steroids and their epithelial influence

Principal component analysis (PCA) was used to evaluate the impact of oral steroid therapy on gene expression (S4 Fig). The patient samples did not form distinct clusters on the PCA plot based on the type of therapy they were being administered, suggesting no distinction in gene expression profiles of patients on oral steroids versus those on high dose inhaled steroids only. Differential gene expression analysis did not reveal any added impact of oral steroid therapy, with only 1 gene differentially expressed in peripheral airways (*NRIP3*) and 3 genes differentially expressed in central airways (*DEFA1*, *BDNF*, *STARD5*) between patients on oral steroids and those on high dose inhaled steroids only. However, there was an imbalance between the number of patients on oral steroids and those on high dose inhaled steroids only (Table 2) as this study was not designed to specifically evaluate the impact of oral steroid therapy on gene expression.

## Discussion

**Fig 5:**
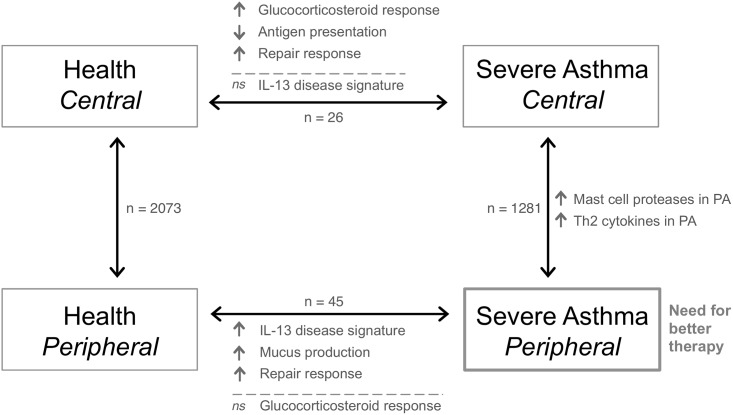
Schematic depicting the conclusions from the study. The main findings along with the numbers of differentially expressed genes are listed for each comparison. *PA*, peripheral airways; *ns*, not significant.

For the first time, peripheral airways in severe asthma have been characterized using a transcriptomics approach and compared to the more traditionally studied central airways. A persistent disease signature associated with IL-13 (*CLCA1*, *POSTN*, *SERPINB2*) was identified in peripheral airways in treatment-resistant severe asthma. The IL-13 signature (*CLCA1*, *POSTN*, *SERPINB2*) was previously described by Woodruff et al. [5] as being upregulated in epithelial brushings from central airways of milder asthmatics but subsequently downregulated by glucocorticosteroid therapy. The significant upregulation of these IL-13 response genes in the peripheral airways in this study suggests that glucocorticosteroids are ineffective at downregulating these genes in peripheral airways of severe asthmatics. A lack of effect that putatively may relate to ineffective target dose delivery or local factors modifying the biological response. This potential lack of efficacy of glucocorticosteroids is further implied as *FKBP5*, a common molecular marker of glucocorticosteroid response [5], failed to attain significance in peripheral airways when severe asthmatics were compared to healthy volunteers. Conversely, in central airways the IL-13 response signature was diminished in severe asthmatics compared to peripheral airways and failed to attain significance (Fig 2B). Additionally, *FKBP5* was significantly upregulated in the central airways in severe asthma, indicative of a strong response to glucocorticosteroids at this large central airways site. Thus, in treatment-resistant severe asthma there appears to be an effective steroid response in central airways, which is less evident in peripheral airways where the IL-13 disease signature persists (Fig 5). This would be consistent with recently reported central airway biopsy findings in severe asthma [29].

The diminished glucocorticosteroid response in peripheral airways was observed despite high dose inhaled steroid therapy, including the use of ultrafine particle inhalers and additional treatment with oral steroids in 4 patients. Previous studies with radiolabelled aerosols reveal that majority of the inhaled steroid therapy is deposited within central airways [30]. Although the use of ultrafine particle inhaler therapy increases peripheral airway deposition, only 15–20% of the prescribed dose reaches these smaller peripheral airways upon inhalation [31], which account for 98% of the airway surface area and thus the major component of the lung [8]. As such, due to the substantial difference in surface area, the local concentration on peripheral airway epithelial cells will be considerably less than that within the central airways. Further, perceived inability of glucocorticosteroids to modify the IL-13 disease signature in peripheral airways cannot be explained by lack of adherence to therapy, as there was effective delivery observed in paired samples from central airways of the same patients. Whilst microarray analysis and RT-qPCR identified some expression of *FKBP5* within the peripheral airways, this was much lower than the expression in the central airways in severe asthma and not significantly different from healthy controls (Fig 2B and 2C). Moreover, the administration of oral steroids did not reveal a distinct gene expression signature (S4 Fig). This finding, suggestive of a lack of effect on the epithelial activation status over and above that of inhaled steroids, is not inconsistent with the previous reported finding that oral steroids alone do not reduce epithelial mast cell numbers [32]. Future studies, however, with larger balanced numbers, need to be carried out to comprehensively evaluate the differences between the effects of oral steroids versus the effects of inhaled steroids on epithelial gene expression. Whist it is now well established that asthma is a heterogeneous disease [7]. This was evident in the severe asthma samples analysed in this study (Fig 4), it was observed that there was a poor response to steroid therapy in the peripheral airways across all patients. Thus this gene expression signal suggesting a lack of therapeutic modification by steroids within the peripheral airways is likely to be a significant contributor to treatment-resistant disease and could arise either on account of inadequate local steroid concentration on the epithelial surface or local factors within the peripheral airways modifying the glucocorticosteroid responsiveness.

Direct comparison between the airways within severe asthma identified greater expression of mast cells proteases (*CPA3*, *TPSB2*, *TPSAB1*) in the peripheral airways (Fig 5). This is in concord with the observations from Balzar et al. [13] who reported increased mast cells in transbronchial biopsies obtained from small airway lung region compared with large airways. Other studies have reported increased levels of mast cell related enzyme, *PGD2* [33] and increased numbers of metachromatic cells [34] in central airways as being correlated with asthma severity. The mean inhaled steroid dose in this quoted study was, however, less than half the mean steroid dose of the severe asthmatics studied in the present study. The absence of mast cell accumulation in central airways in our study is thus likely to reflect the more intense therapy, as topical steroid delivery is known to reduce intraepithelial mast cell accumulation [35]. Of relevance, activated mast cells have relevance to the pathophysiology of asthma [36] and represent a cellular source of Th2 cytokines. Consistent with this, is the increased expression of *CLCA1* and *interleukin 1 receptor-like 1* (*IL1RL1*) in peripheral airways (S1 Table). *ILIRL1* is associated with Th2 inflammation [37] as well as goblet cell hyperplasia [38], which is in concord with the increased mucus production in the peripheral airways in severe asthma in our study, reflected by markers of mucus production, *mucin 5AC* (*MUC5AC*) and *mucin 6* (*MUC6*) that had a greater expression in severe asthma compared to health in peripheral airways but failed to attain significance (nominal p-value < 0.05; data not shown). Moreover, Secretoglobin, Family 1A, Member 1 (*SCGB1A1*), which is often used as a gene expression marker of Club cell, was negatively downregulated in severe asthma compared to health in both central airways and in peripheral airways (S3 Table). The negative down regulation of the secretoglobin family in asthma is in line with previously published studies [5]. Thus the identification of persistent intraepithelial mast cell presence and abnormal Type2 signalling within the peripheral airways reveals the presence of abnormal biology of relevance to the pathophysiology of severe asthma that is not supressed by current therapy.

An altered epithelial repair response was observed across both airways in severe asthma, evident by the increased expression of cytokeratin genes such as *FGFBP1* (S3 Table). *FGFBP1* encodes a secreted fibroblast growth factor carrier protein that binds to fibroblast growth factor (*FGF*) and potentiates its effect in promoting biological effect on mesenchymal cells and in epithelial repair [39]. The availability of *FGF* has been linked to mast cell activation through the release of heparin and endoglycosidases cleaving *FGF* from its proteoglycan binding [40]. Thus intra-epithelial mast cell presence has relevance to the local release of *FGF* in peripheral airways and its ability to interact with *FGFBP1*. An altered repair response is further reflected by increased epithelial cell recovery in BAL from the severe asthma, as compared to that in the healthy subjects (Table 1). Epithelial brushings, whilst mainly representing airway epithelial cells will also contain cells that have infiltrated into the airway epithelial compartment. As such, although the gene signature will be predominantly derived from epithelial cell activation there may be a mixed sample source and the results should be interpreted carefully as the cellular composition could vary between samples. Furthermore, the statistical filtering approach utilized in this study based on FDR corrected p-values, although common practice, is based on arbitrary cut-offs and should be interpreted with this consideration in mind. There have been studies that have utilized similar sample sources, such as the landmark study by Woodruff et al. [5] that successfully established the bronchial epithelial IL-13 disease signature. Our study has extended such an approach to further our understanding of the peripheral airways in severe asthma. There are several tools that are now available for cell-type enrichment analysis to identify cellular composition on a per sample basis. However, such approaches have not been completely established yet and depend heavily on marker genes that are used [41, 42], which are not readily available for the cell types present in epithelial brushings. Future evaluations in pure cell populations will help further characterize the role of peripheral airways in severe asthma.

## Conclusions

In summary, global gene expression profiling of epithelial brushings using microarrays was used to comprehensively characterize the phenotype of peripheral airways in severe asthma in an unbiased approach (Fig 5). To our knowledge, this is the first such study investigating the epithelial biology of peripheral airways as a consequence of severe asthma at the transcriptome level. Our findings suggest a crucial role of peripheral airways in disease pathogenesis and disease related changes persist in the peripheral airways of treatment-resistant severe asthmatics despite the administration of high doses of current anti-inflammatory asthma therapy. This suggests that severe asthma is a disease that includes a significant peripheral airway component and as such, much information relevant to the disease process has previously been overlooked when only the central airways have been assayed. Future studies in mild and moderate asthma will inform as to the extent to which the IL13 disease signature is evident in the peripheral airways throughout the entire spectrum of this disease and its relationship to disease control. In conclusion, the present findings in treatment-resistant severe asthma suggest a need for disease modifying therapy that is effective within the small peripheral airways.

## Supporting Information

S1 Fig Three-dimensional principal component analysis representing two batches in the microarray data. **A.** before and **B.** after correction. Each circle represents a sample that is coloured red or black based on the year of microarray hybridization. Different sample types were approximately equally distributed across the two batches. Fourteen central airway samples in health, 10 peripheral airway samples in health, 9 central airway samples in severe asthma and 11 peripheral airway samples in severe asthma were present in batch 1, and 9, 9, 4 and 4 samples respectively were present in batch 2. *PC1*, *PC2* and *PC3* represent the first, second and third principal components respectively.
(TIF)

S2 Fig Three-dimensional principal component analysis representing the smoking status of subjects who participated in the study. Each circle represents a sample that is coloured red for non-smokers and black for ex-smokers. *Comp1*, *Comp2* and *Comp3* represent the first, second and third principal components respectively.
(TIFF)

S3 Fig Three-dimensional principal component analysis examining the effect of disease status and airway type. Each circle represents a sample that is coloured black for central airways in health, red for peripheral airways in health, green for central airways in severe asthma and blue for peripheral airways in severe asthma. *Comp1*, *Comp2* and *Comp3* represent the first, second and third principal components respectively.
(TIF)

S4 Fig Principal component analysis to evaluate the impact of oral steroid therapy on gene expression in severe asthmatics. Samples from both central airways and peripheral airways from severe asthmatics are depicted. Circles represent inhaled corticosteroids only and triangles represent oral corticosteroids in addition to inhaled corticosteroids. *PC1* and *PC2* represent the first and second principal components respectively.
(TIF)

S1 File Supporting information file that contains supplementary methods and results. (PDF)

S1 Table Differential gene expression between peripheral and central airways within health and within severe asthma. A positive fold change indicates higher expression of the gene in peripheral airways compared to central airways and a negative fold change reflects lower expression in peripheral airways compared to central airways. Significant values are presented in bold font.
(XLSX)

S2 Table Functional enrichment for the gene modules identified using SSIM for the list of genes differentially expressed between peripheral and central airways, uniquely severe asthmatics. (XLSX)

S3 Table Differential gene expression between severe asthma and health within central airways and within peripheral airways. A positive fold change indicates higher expression of the gene in severe asthma compared to health and a negative fold change reflects lower expression in severe asthma. Significant values are presented in bold font.
(XLSX)

## References

[1] Bateman ED, Boushey HA, Bousquet J, Busse WW, Clark TJ, Pauwels RA, et al Can guideline-defined asthma control be achieved? The Gaining Optimal Asthma ControL study. Am J Respir Crit Care Med. 2004;170(8):836–44. 10.1164/rccm.200401-033OC 15256389

[2] Montuschi P, Barnes PJ. New perspectives in pharmacological treatment of mild persistent asthma. Drug Discov Today. 2011;16(23):1084–91. 21930234 10.1016/j.drudis.2011.09.005

[3] Holgate ST, Holloway J, Wilson S, Bucchieri F, Puddicombe S, Davies DE. Epithelial–mesenchymal communication in the pathogenesis of chronic asthma. Proceedings of the American Thoracic Society. 2004;1(2):93–8. 10.1513/pats.2306034 16113419

[4] Loxham M, Davies DE, Blume C. Epithelial Function and Dysfunction in Asthma. Clinical & Experimental Allergy. 2014;44(11):1299–313. 24661647 10.1111/cea.12309

[5] Woodruff PG, Boushey HA, Dolganov GM, Barker CS, Yang YH, Donnelly S, et al Genome-wide profiling identifies epithelial cell genes associated with asthma and with treatment response to corticosteroids. Proceedings of the National Academy of Sciences. 2007;104(40):15858–63. 10.1073/pnas.0707413104 PMC2000427 17898169

[6] Woodruff PG, Modrek B, Choy DF, Jia G, Abbas AR, Ellwanger A, et al T-helper type 2–driven inflammation defines major subphenotypes of asthma. Am J Respir Crit Care Med. 2009;180(5):388–95. 10.1164/rccm.200903-0392OC 19483109 PMC2742757

[7] Wenzel SE. Asthma phenotypes: the evolution from clinical to molecular approaches. Nat Med. 2012;18(5):716–25. 10.1038/nm.2678 22561835

[8] Weibel ER. Morphometry of the human lung: Springer; 1965.

[9] van der Wiel E, ten Hacken NH, Postma DS, van den Berge M. Small-airways dysfunction associates with respiratory symptoms and clinical features of asthma: a systematic review. J Allergy Clin Immunol. 2013;131(3):646–57. 10.1016/j.jaci.2012.12.1567 23380222

[10] Riley CM, Wenzel SE, Castro M, Erzurum SC, Chung KF, Fitzpatrick AM, et al Clinical Implications of Having Reduced Mid Forced Expiratory Flow Rates (FEF25-75), Independently of FEV1, in Adult Patients with Asthma. PLoS One. 2015;10(12). 10.1371/journal.pone.0145476 PMC4696666 26717486

[11] Tulic MK, Christodoulopoulos P, Hamid Q. Small airway inflammation in asthma. Respiratory research. 2001;2(6):333 10.1186/rr83 11737932 PMC64806

[12] Andersson CK, Bergqvist A, Mori M, Mauad T, Bjermer L, Erjefält JS. Mast cell–associated alveolar inflammation in patients with atopic uncontrolled asthma. J Allergy Clin Immunol. 2011;127(4):905–12.e7. 10.1016/j.jaci.2011.01.022 21388666

[13] Balzar S, Chu HW, Strand M, Wenzel S. Relationship of small airway chymase-positive mast cells and lung function in severe asthma. Am J Respir Crit Care Med. 2005;171(5):431–9. 10.1164/rccm.200407-949OC 15563633

[14] Howarth P. Small airway inflammation and asthma. International journal of clinical practice Supplement. 1998;96:15–22. 10344029

[15] Hamid Q, Song Y, Kotsimbos TC, Minshall E, Bai TR, Hegele RG, et al Inflammation of small airways in asthma. J Allergy Clin Immunol. 1997;100(1):44–51. 9257786 10.1016/s0091-6749(97)70193-3

[16] Minshall EM, Hodd JC, Hamid QA. Cytokine mRNA expression in asthma is not restricted to the large airways. J Allergy Clin Immunol. 1998;101(3):386–90. 9525456 10.1016/s0091-6749(98)70252-0

[17] Taha RA, Minshall EM, Miotto D, Shimbara A, Luster A, Hogg JC, et al Eotaxin and monocyte chemotactic protein-4 mRNA expression in small airways of asthmatic and nonasthmatic individuals. J Allergy Clin Immunol. 1999;103(3):476–83. 10069883 10.1016/s0091-6749(99)70474-4

[18] Wu Z, Irizarry RA, Gentleman R, Martinez-Murillo F, Spencer F. A model-based background adjustment for oligonucleotide expression arrays. Journal of the American statistical Association. 2004;99(468):909–17.

[19] Woelk CH, Zhang JX, Walls L, Viriyakosol S, Singhania A, Kirkland TN, et al Factors regulated by interferon gamma and hypoxia-inducible factor 1A contribute to responses that protect mice from Coccidioides immitis infection. BMC Microbiol. 2012;12(1):218. 23006927 10.1186/1471-2180-12-218 PMC3528620

[20] Ringnér M. What is principal component analysis? Nat Biotechnol. 2008;26(3):303–4. 10.1038/nbt0308-303 18327243

[21] Leek JT, Johnson WE, Parker HS, Jaffe AE, Storey JD. SVA: surrogate variable analysis. R package version. 2013;3(0).

[22] Smyth GK. Limma: linear models for microarray data Bioinformatics and computational biology solutions using R and Bioconductor: Springer; 2005 p. 397–420.

[23] Chen J, Bardes EE, Aronow BJ, Jegga AG. ToppGene Suite for gene list enrichment analysis and candidate gene prioritization. Nucleic Acids Res. 2009;37(suppl 2):W305–W11. 19465376 10.1093/nar/gkp427 PMC2703978

[24] Supek F, Bošnjak M, Škunca N, Šmuc T. REVIGO summarizes and visualizes long lists of gene ontology terms. PloS one. 2011;6(7):e21800 10.1371/journal.pone.0021800 21789182 PMC3138752

[25] Cho J-H, Wang K, Galas DJ. An integrative approach to inferring biologically meaningful gene modules. BMC systems biology. 2011;5(1):117. 21791051 10.1186/1752-0509-5-117 PMC3156758

[26] Massanella M, Singhania A, Beliakova-Bethell N, Pier R, Lada SM, White CH, et al Differential gene expression in HIV-infected individuals following ART. Antiviral Res. 2013;100(2):420–8. 10.1016/j.antiviral.2013.07.017 23933117 PMC3899918

[27] Benjamini Y, Hochberg Y. Controlling the false discovery rate: a practical and powerful approach to multiple testing. Journal of the Royal Statistical Society Series B (Methodological). 1995:289–300.

[28] Li H, Wood CL, Getchell TV, Getchell ML, Stromberg AJ. Analysis of oligonucleotide array experiments with repeated measures using mixed models. BMC Bioinformatics. 2004;5(1):1. 15626348 10.1186/1471-2105-5-209 PMC544885

[29] Wilson SJ, Ward JA, Sousa AR, Corfield J, Bansal AT, De Meulder B, et al Severe asthma exists despite suppressed tissue inflammation: findings of the U-BIOPRED study. Eur Respir J. 2016;48(5):1307–19. 10.1183/13993003.01129-2016 27799384

[30] Laube B, Swift D, Wagner H Jr, Norman P, Adams G 3rd. The effect of bronchial obstruction on central airway deposition of a saline aerosol in patients with asthma. The American review of respiratory disease. 1986;133(5):740–3. 3706880 10.1164/arrd.1986.133.5.740

[31] Leach CL, Bethke TD, Boudreau RJ, Hasselquist BE, Drollmann A, Davidson P, et al Two-dimensional and three-dimensional imaging show ciclesonide has high lung deposition and peripheral distribution: a nonrandomized study in healthy volunteers. Journal of aerosol medicine. 2006;19(2):117–26. 10.1089/jam.2006.19.117 16796536

[32] Djukanović R, Homeyard S, Gratziou C, Madden J, Walls A, Montefort S, et al The effect of treatment with oral corticosteroids on asthma symptoms and airway inflammation. Am J Respir Crit Care Med. 1997;155(3):826–32. 10.1164/ajrccm.155.3.9117012 9117012

[33] Fajt ML, Gelhaus SL, Freeman B, Uvalle CE, Trudeau JB, Holguin F, et al Prostaglandin D2 pathway upregulation: Relation to asthma severity, control, and TH2 inflammation. J Allergy Clin Immunol. 2013;131(6):1504–12. e12. 10.1016/j.jaci.2013.01.035 23506843 PMC3889167

[34] Gibson PG, Saltos N, Borgas T. Airway mast cells and eosinophils correlate with clinical severity and airway hyperresponsiveness in corticosteroid-treated asthma. J Allergy Clin Immunol. 2000;105(4):752–9. 10.1067/mai.2000.105319 10756226

[35] Bradding P, Feather IH, Wilson S, Holgate ST, Howarth PH. Cytokine immunoreactivity in seasonal rhinitis: regulation by a topical corticosteroid. Am J Respir Crit Care Med. 1995;151(6):1900–6. 10.1164/ajrccm.151.6.7767538 7767538

[36] Marone G, Triggiani M, Genovese A, Paulis AD. Role of human mast cells and basophils in bronchial asthma. Adv Immunol. 2005;88:97–160. 10.1016/S0065-2776(05)88004-6 16227089

[37] Traister RS, Uvalle CE, Hawkins GA, Meyers DA, Bleecker ER, Wenzel SE. Phenotypic and genotypic association of epithelial IL1RL1 to human T H 2-like asthma. J Allergy Clin Immunol. 2015;135(1):92–9. e10. 10.1016/j.jaci.2014.06.023 25091434 PMC4289095

[38] Schmitz N, Kurrer M, Kopf M. The IL-1 receptor 1 is critical for Th2 cell type airway immune responses in a mild but not in a more severe asthma model. Eur J Immunol. 2003;33(4):991–1000. 10.1002/eji.200323801 12672065

[39] Beer H-D, Bittner M, Niklaus G, Munding C, Max N, Goppelt A, et al The fibroblast growth factor binding protein is a novel interaction partner of FGF-7, FGF-10 and FGF-22 and regulates FGF activity: implications for epithelial repair. Oncogene. 2005;24(34):5269–77. 10.1038/sj.onc.1208560 15806171

[40] Shute J, Solic N, Shimizu J, McConnell W, Redington A, Howarth P. Epithelial expression and release of FGF-2 from heparan sulphate binding sites in bronchial tissue in asthma. Thorax. 2004;59(7):557–62. 10.1136/thx.2002.002626 15223860 PMC1747073

[41] Newman AM, Liu CL, Green MR, Gentles AJ, Feng W, Xu Y, et al Robust enumeration of cell subsets from tissue expression profiles. Nat Methods. 2015;12(5):453–7. 10.1038/nmeth.3337 25822800 PMC4739640

[42] Shen-Orr SS, Gaujoux R. Computational deconvolution: extracting cell type-specific information from heterogeneous samples. Curr Opin Immunol. 2013;25(5):571–8. 10.1016/j.coi.2013.09.015 24148234 PMC3874291

