# Supplementary figures and images for: Altered Epithelial Gene Expression in Peripheral Airways of Severe Asthma
Source: PLoS One. 2017 Jan 3;12(1):e0168680. doi: 10.1371/journal.pone.0168680 (PMC5207492; doi:10.1371/journal.pone.0168680)

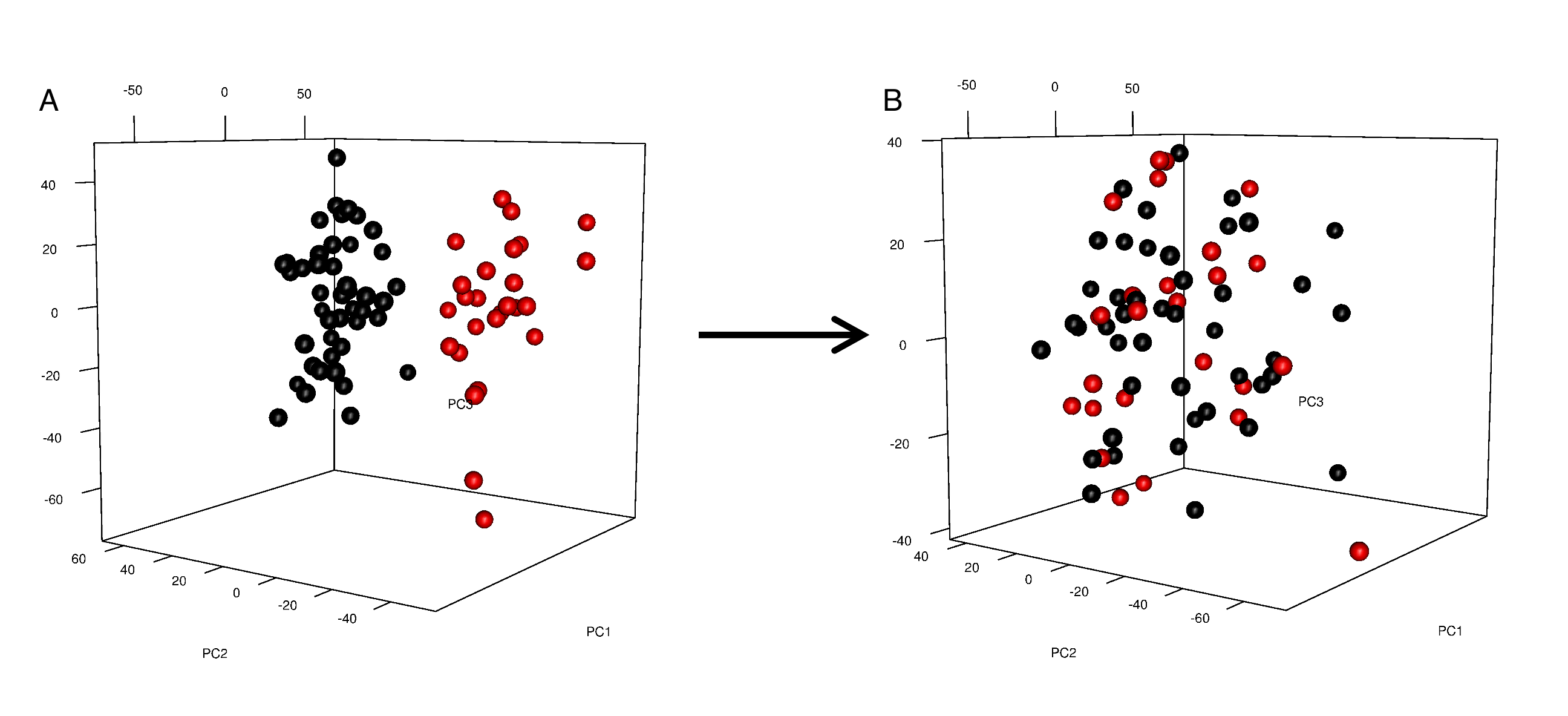

Supplement: S1 Fig — A. before and B. after correction. Each circle represents a sample that is coloured red or black based on the year of microarray hybridization. Different sample types were approximately equally distributed across the two batches. Fourteen central airway samples in health, 10 peripheral airway samples in health, 9 central airway samples in severe asthma and 11 peripheral airway samples in severe asthma were present in batch 1, and 9, 9, 4 and 4 samples respectively were present in batch 2. PC1, PC2 and PC3 represent the first, second and third principal components respectively. (TIF) [file pone.0168680.s001.tif]

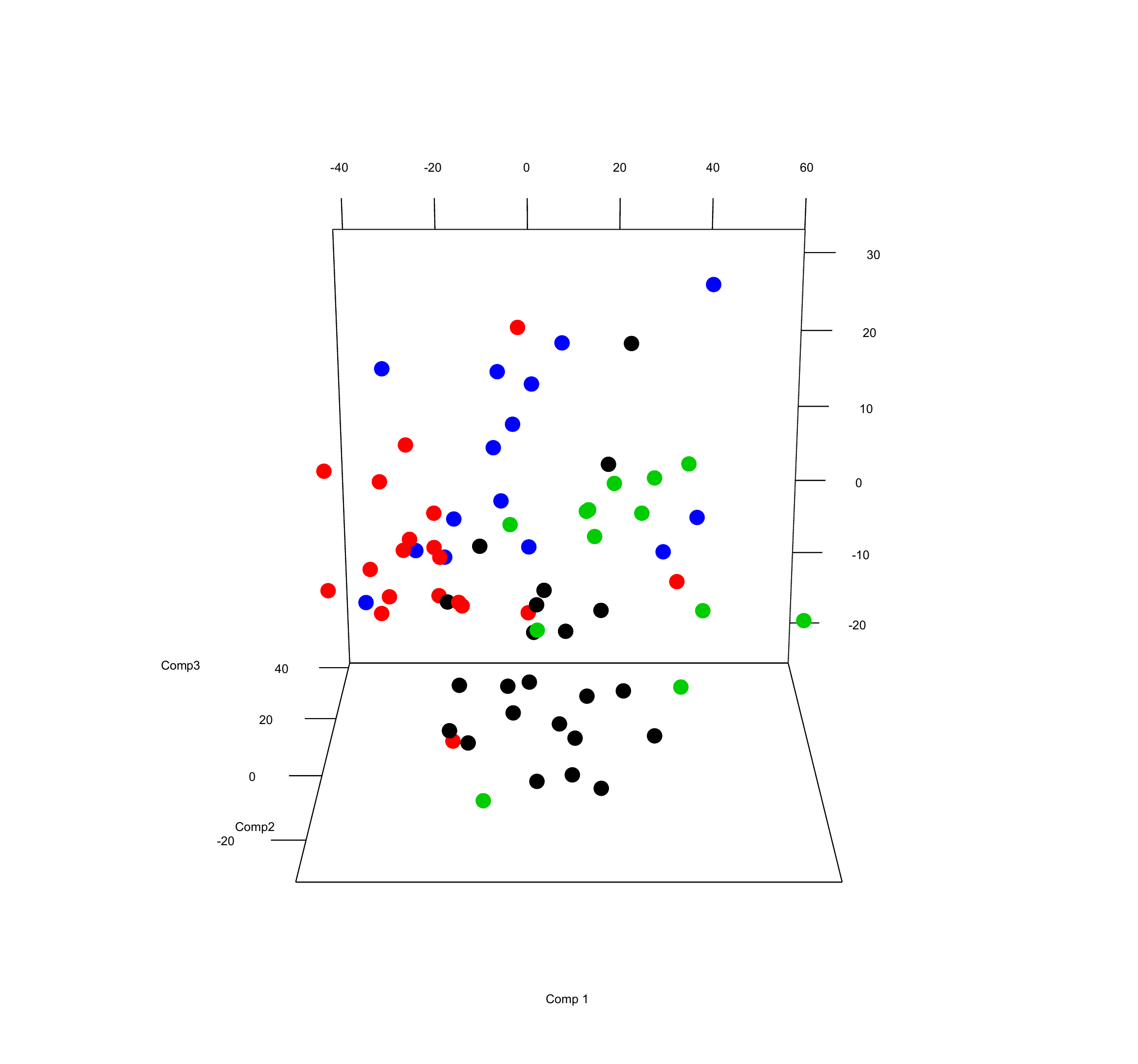

Supplement: S3 Fig — Each circle represents a sample that is coloured black for central airways in health, red for peripheral airways in health, green for central airways in severe asthma and blue for peripheral airways in severe asthma. Comp1, Comp2 and Comp3 represent the first, second and third principal components respectively. (TIF) [file pone.0168680.s003.tif]

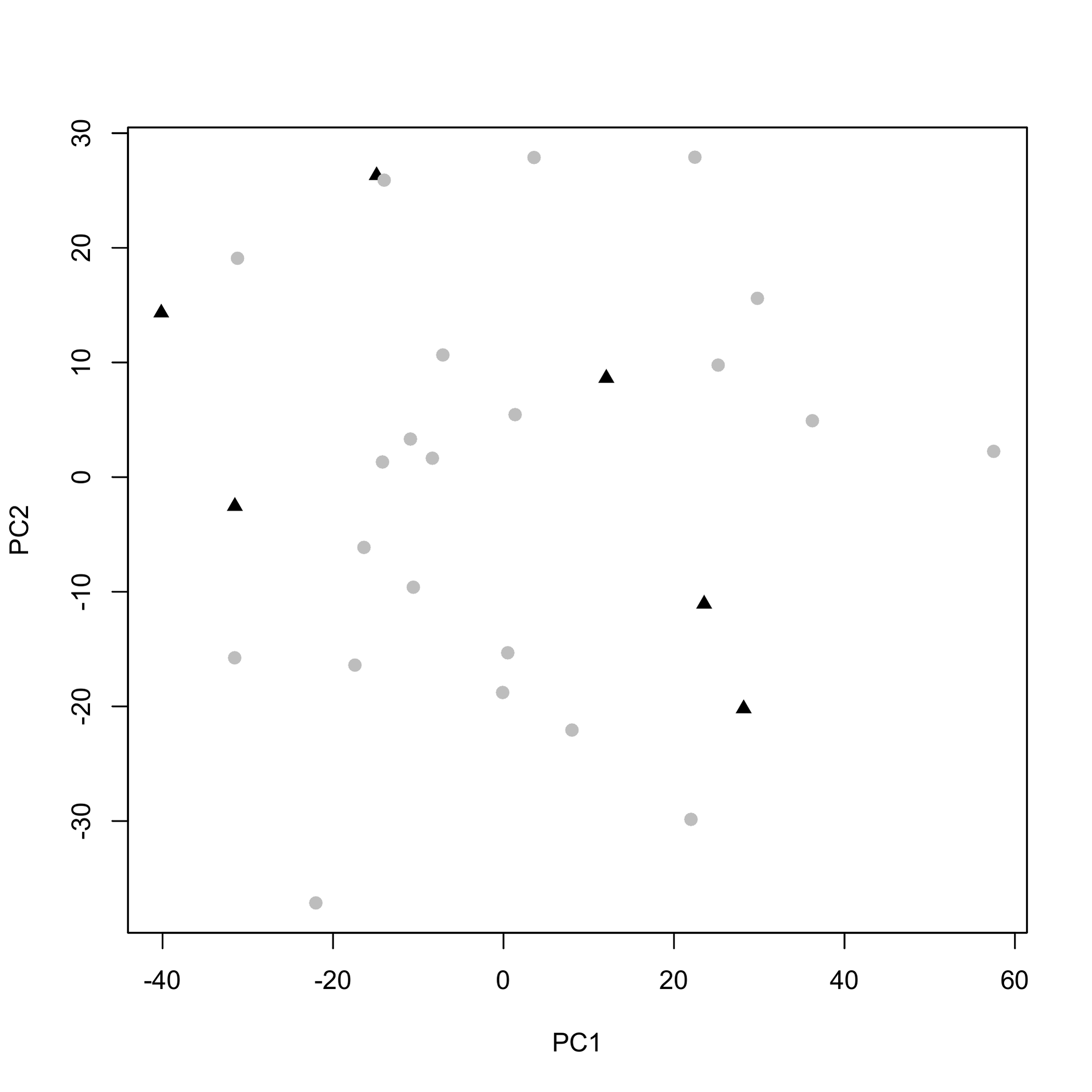

Supplement: S4 Fig — Samples from both central airways and peripheral airways from severe asthmatics are depicted. Circles represent inhaled corticosteroids only and triangles represent oral corticosteroids in addition to inhaled corticosteroids. PC1 and PC2 represent the first and second principal components respectively. (TIF) [file pone.0168680.s004.tif]
