# Supplementary material for: Altered Epithelial Gene Expression in Peripheral Airways of Severe Asthma
Source: PLoS One. 2017 Jan 3;12(1):e0168680. doi: 10.1371/journal.pone.0168680 (PMC5207492; doi:10.1371/journal.pone.0168680)
Supplement: S1 File — (PDF) [file pone.0168680.s005.pdf]

Supporting Information

Altered Epithelial Gene Expression in Peripheral Airways of Severe Asthma

Akul Singhania[1], Hitasha Rupani[1], Nivenka Jayasekera[1], Simon Lumb[2], Paul Hales[2], Neil Gozzard[2], Donna E. Davies[1,3], Christopher H. Woelk[1, ¶,*], Peter H. Howarth[1,3, ¶,*]

[1]Clinical and Experimental Sciences, Faculty of Medicine, University of Southampton, Southampton, SO16 6YD, UK

[2]UCB Celltech, 216 Bath Road, Slough SL1 3WE

[3]Southampton NIHR Respiratory Biomedical Research Unit, Southampton Centre for Biomedical Research, University Hospital Southampton NHS Foundation Trust, Southampton, SO16 6YD, UK

*Corresponding authors

E-mail: c.h.woelk@soton.ac.uk (CHH)

E-mail: p.h.howarth@soton.ac.uk (PHH)

[¶]These authors contributed equally to this work.

Table Of Contents

Additional Methodological Information **3**

Dates of study 3

Study volunteers 3

Spirometry 4

Atopy assessment 5

Bronchoscopy 5

Bronchoalveolar lavage 6

Bronchial epithelial brushings 6

RNA isolation and microarray hybridization 7

Microarray Data Analysis 7

Real-time quantitative PCR (RT-qPCR) analysis 9

Additional Discussion Points **10**

Nitric oxide in severe asthma 10

Glucocorticoid effects within central airway in severe asthma 11

Oral steroids and their epithelial influence 11

Epithelial genes and exacerbation frequency 12

References **13**

Additional Methodological Information

Dates of study

The samples were collected as part of a basic research study in asthma that received ethical approval on 21/03/2006 (REC reference number 05/Q1702/165). The study samples were collected between 12/05/2010 and 13/3/2012. Data acquisition was achieved by 05/09/2012 and initial analysis was completed in 20/12/2014.

Study volunteers

Adult volunteers aged 18 to 65 years were recruited from the Wessex Severe Asthma Cohort and from a departmental database of volunteers (**Table 1**). The study was approved by the local research ethics committee and all subjects gave written informed consent. Adults with no history of respiratory disease, no current symptoms and no drop in forced expiratory volume in one second (FEV_1) with inhaled methacholine at 16mg/mL were defined as healthy controls. Three had been ex-smokers with a pack year history of <2 and had ceased smoking for at least 1 year. The severe asthma patients were on step 4 or 5 of GINA guidelines therapy (1), had poorly controlled disease with an Asthma Control Questionnaire (ACQ) score of ≥1.5 (mean 3.09 range 1.71-4.86) and fulfilled the International ERS/ATS criteria for severe asthma (2). All were lifelong non-smokers with the exception of two who had abstained from smoking for 7 and 29 years (5 and 17 pack years respectively). They were on a mean daily inhaled steroid dose (beclometasone equivalents) of 2729

micrograms (range 2000-4400 μg) (**Table 1** in main text). Four were also on additional daily oral steroids, mean daily dose 11.25 mg (range 5 mg to 15mg with the one receiving the lowest oral steroid dose also being on weekly methotrexate 10mg). All were on daily therapy with inhaled long acting beta agonists. All had also received at some time oral therapy with montelukast 10mg nocte, with 10 currently on this treatment. All the severe asthmatics had a history of at least 2 exacerbations in the last year (median 4, range 2-12) and 44% had a history of hospital admission for acute severe asthma in the last year.

Seventeen severe asthma patients were enrolled in the microarray study (central airways samples obtained from 13 patients and peripheral airways samples obtained from 15 patients – with 11 patients with paired samples from both airways) and 23 non-asthmatic healthy volunteers (central airways samples obtained from 23 volunteers and peripheral airways samples obtained from 19 volunteers – with 19 volunteers with paired samples from both airways) (**Table 2** in main text). Additional assessments as markers of airway inflammation were measurement of fraction of nitric oxide in exhaled air (FeNO) and luminal airway inflammatory cell profile, as assessed by bronchoalveolar lavage (BAL) cytospin differential cell count (**Table 1** in main text). FeNO was measured with either the NIOX analyser or NIOX MINO (Aerocrine AB, Solna, Sweden) according to guidelines (3) at an exhaled flow rate of 50 mL/second. For each patient at least three correctly executed exhalations with the device were required and the mean value was recorded. BAL sampling details are given in Section 1.6.

Spirometry

Spirometry was performed with a dry bellows spirometer (Vitalograph, UK) and the best of at least three successive readings within 100 ml of each other was recorded as the FEV_1. Measures were made before and 15 minutes after the administration of nebulised salbutamol (2.5mg).

Atopy assessment

Skin prick testing on the volar surface of the forearm was performed using the following eight allergen extracts (Allergopharma, Germany): *aspergillus fumigatus, alternaria tenius*, birch tree pollen, mixed grasses, *dermatophagoides pteronyssinus, dermatophagoides farinae*, dog fur and cat dander, along with positive (histamine) and negative (saline) controls. A positive allergen response was taken as a skin wheal response of 3mm or greater than the saline negative control. An individual was defined as atopic if they had one or more positive allergen skin wheal responses. The absence of any positive allergen response in the presence of a positive histamine skin wheal response was indicative of non-atopic status.

Bronchoscopy

Fibre optic bronchoscopy was performed according to British Thoracic Society (BTS) guidelines (4) and the local departmental standard operating procedure in the NIHR Respiratory Bioscience Research Unit (RBRU), which is part of the Southampton Centre for Biomedical Research (SCBR) at Southampton General Hospital.

Bronchoalveolar lavage

To assess luminal airway inflammation, BAL was performed by wedging the bronchoscope in a right upper lobe subsegmental bronchus and instilling 6x20 ml aliquots of pre warmed normal saline followed by gentle suction. Recovered BAL fluid was filtered (BD Falcon cell strainer, Marathon Laboratory Supplies, UK) then centrifuged at 1300G for 10 mins at 4°C. The supernatant was aliquoted, prior to storage at -80°C, and the cell pellet resuspended in phosphate buffered saline, cytocentrifuged (Thermo Shandon Ltd, UK) and the slides stained with rapid Romanowsky stain (Raymond Lamb Ltd, UK) for differential cell counts. A total of 400 cells were counted on coded samples in a blinded manner.

Bronchial epithelial brushings

Epithelial brushings were collected using disposable, sheathed bronchial brushes (Olympus cytology brushes BC-202D-1210, KeyMed [Medical & Industrial Equipment] Ltd OLYMPUS Group Company), with 4 separate sets of brushings from the central airways and 4 from the peripheral airways. Central brushes were taken under direct vision from the right bronchus intermedius by gently rubbing the unsheathed brush against the mucosal surface. Peripheral brushings were obtained by extending the sheathed brush (external diameter 1.2 mm) out of vision as far as possible into a right lower lobe sub-segmental bronchus, until an appreciation of localised awareness by the volunteer suggestive of a pleural response, followed by slight retraction and then extension of the brush (diameter 0.064 mm) beyond the sheath to obtain the epithelial brush samples. The bronchial brushings were placed

in sterile universal tubes with 5 ml PBS (4 brushings per tube), with the central and peripheral brushings collected separately. Five ml of RPMI medium (Invitrogen, UK) (with 1% Pen/strep, 1% L-glutamine and 20% FBS) was added and the tubes spun at 1000 rpm for 5 minutes to pellet the cells. The medium was discarded and 1 ml of RNA later Reagent (Invitrogen, Paisley, UK) was added to the cell pellet in each universal tube, followed by pipetting up and down to solubilise the cells and incubation at room temperature for 5 minutes. The homogenate was transferred to 1500ul RNAse- and DNAse-free Eppendorf microtubes (Sigma-Aldrich, Gillingham, Dorset, UK) for storage at -80^{0}C prior to RNA extraction.

RNA isolation and microarray hybridization

RNA was isolated from the samples using the Qiagen miRNeasy Kit (Qiagen Ltd., Manchester, UK Cat No: 217004) and the RNA quality was assessed using a Bioanalyzer 2100 (Agilent Ltd., Cheshire, UK). Samples were hybridized to Affymetrix HG U133 plus 2.0 beadchips (Affymetrix, Santa Clara, California, USA) by Almac Diagnostics ltd., Craigavon, Northern Ireland. Gene expression data are available at the Gene Expression Omnibus (http://www.ncbi.nlm.nih.gov/geo) under accession number GSE64913.

Microarray Data Analysis

Raw microarray gene expression data was normalized using Gene Chip Robust Multi-Array Analysis (GCRMA) (5) using the *bioconductor* (version 2.13) package *gcrma* in R (version 3.0.2) and subjected to several quality control

procedures such as boxplots, histograms, unsupervised clustering approaches and interquartile range vs. median plots. Principal component analysis (PCA) revealed two distinct batches based on the year of microarray hybridization and these batch effects were removed using the sva package (6) as a number of central and peripheral airway samples from health as well as severe asthma were included in both batches (**S1 Figure**). The smoking status of subjects did not have an effect on the gene expression (**S2 Figure**) and all subjects were retained for further analysis. Non-specific filtering of genes was also carried out to remove genes with low mean expression levels and small standard of deviation across samples. Differentially expressed genes were identified using *limma* (7) in R in a linear mixed modelling approach where correlations were computed for subjects with paired samples from both airways sites (11 paired samples in severe asthma and 19 in health, **Table 2** in main text) and included during model fitting to obtain differentially expressed genes between all samples (paired as well as unpaired). Age mismatch between the severe asthma and health group (**Table 1** in main text) was removed during differential expression analysis by fitting age in the linear model. Atopy mismatch between the severe asthma and health group could not be removed using the linear model due to very few health subjects being atopic (**Table 1** in main text), however, comparison between atopic and non-atopic individuals within peripheral and central airways did not generate any differentially expressed genes (data not shown). Only genes with False Discovery Rate (FDR) corrected p-values < 0.05 for multiple testing) were considered significant. The FDR in all analyses was corrected for using the Benjamini-Hochberg (BH) method (8). Gene ontology (GO) analysis was performed using ToppGene in order to identify those GO terms that were significantly overrepresented (FDR correction using BH) for differentially expressed genes (9).

The resulting GO terms were collapsed into categories of related terms using REVIGO (10).

A semantic similarity-integrated approach for modularization (SSIM) was performed (11) in order to find modules of functionally related genes based on co-expression, protein-protein interaction and shared gene ontology. Gene-gene pairwise similarity indices were computed by constructing 3 similarity matrices: (i) expression similarity matrix, by calculating Pearson correlations for gene expression values between all differentially expressed genes, (ii) topological overlap matrix, using combined scores from STRING 9.1 database, which represents the association strength between protein-protein interactions between the protein products of the differentially expressed genes, (iii) GO semantic similarity matrix, by computing GO semantic similarity measures using the Wang method (12) in the *bioconductor* (version 3.0) package *GoSemSim* (13) in R (version 3.1.2). A combined similarity matrix was calculated by converting these individual similarity measures into probability values using the empirical cumulative density function in R and then multiplying these probabilities to obtain a joint probability. Finally, affinity propagation was used to find modules of functionally related genes in the combined similarity matrix using the package *apcluster* (14) in R.

Real-time quantitative PCR (RT-qPCR) analysis

RT-qPCR using TaqMan® Gene Expression Assays (Life Technologies, Carlsbad, California, USA) was used to confirm microarray gene expression on an independent cohort of 5 severe asthmatics and 5 healthy controls. Briefly, RNA was isolated and reverse transcribed into cDNA using the High Capacity cDNA Reverse

Transcription Kit (Life Technologies) with random hexamers following manufacturer's instructions. RT-qPCR was performed for each gene target in duplicate using Taqman Universal PCR Master Mix, No AmpErase UNG on 7900HT Fast Real-Time PCR System (Life Technologies). Thermal cycling conditions were 95˚C for 10 minutes followed by 40 cycles of 95˚C for 15 seconds and 60˚C for 1 minute. Changes in gene expression were calculated using the relative quantification (RQ) method ($2^{-\Delta\Delta CT}$) with *GAPDH* as a normalizer. RQ values were $\log_2$ transformed to satisfy the normality assumption and differences in gene expression between groups assessed with a two-tailed student's t-test, paired for comparisons between peripheral and central airways and unpaired for comparisons between severe asthma and health. RT-qPCR validation was performed for 6 genes – *CPA3*, *TPSB2*, *FKBP5*, *POSTN*, *SERPINB2* and *HLA-DOA* (TaqMan Assay IDs: Hs00157019_m1, Hs02576518_gH, Hs01561006_m1, Hs01566734_m1, Hs01010736_m1, Hs01109372_g1, respectively).

Additional Discussion Points

Nitric oxide in severe asthma

IL-13 has been shown to stimulate nitric oxide (NO) generation by peripheral bronchial epithelial cells when cultured *ex vivo* (15). As such, the reports of higher levels of alveolar NO in severe asthmatics, as compared to that measured in mild to moderate asthma (16, 17), support our direct findings in epithelial brush samples, although not all studies that have measured alveolar NO have identified a link with

disease severity (18). The adults studied in this later publication were, however, less severe than those assessed in the present study.

Glucocorticoid effects within central airway in severe asthma

In central airways the epithelial expression of the chemokine *CCL5* as well as *CXCR7* was significantly suppressed in severe asthma compared to health (**S3 Table**), consistent with effective steroid delivery at this site. Furthermore exhaled nitric oxide measures were not significantly different between the healthy controls and severe asthmatics, further consistent with suppression by therapy (**Table 1** in main text). Moreover *HLA-DOA*, which is expressed on dendritic cells and regulates peptide loading on MHC class II molecules, was significantly decreased within the central airways (**S3 Table**). Whether this reduction is related to the disease or the steroid therapy cannot be defined from this study.

Oral steroids and their epithelial influence

Comparison of the peripheral airway epithelial gene expression between those with severe asthma on both inhaled and oral steroid therapy and those severe asthma patients solely on high dose inhaled steroids, did not reveal any added impact of the oral steroid therapy of significance (**Figure 3** in main text). Differential gene expression only identified one gene (*NRIP3*) as differentially expressed in peripheral airways. Whilst systemic steroids have a prominent effect at reducing bone marrow eosinophil progenitor production and thereby reduce circulating

eosinophils and airway eosinophil recruitment, they do not have such a great impact on epithelial mast cell recruitment and by inference epithelial activation. In a placebo controlled study of oral steroids in milder asthma, there was no effect of oral steroids on epithelial mast cell numbers whilst having profound effects on sub-mucosal inflammation (19). By contrast, inhaled and intranasal steroids have a prominent impact on epithelial mast cell accumulation indicative of the importance of the topical effect to achieve this impact (20, 21). Within the central airways, 3 genes were differentially expressed in severe asthmatics on oral steroids compared to those only on inhaled steroids (*DEFA1*, *BDNF* and *STARD5*). However, due to the imbalance in the number of patients on oral steroids and those on high dose inhaled steroids only (**Table 2** in main text), these results must be interpreted carefully until future studies can be carried out on larger balanced numbers.

Epithelial genes and exacerbation frequency

The severe asthma population comprised asthmatics with a tendency for disease exacerbation, in that all gave a history of at least 2 exacerbations in the last year (median 4, range 2-12) and 44% had a history of hospital admission for acute severe asthma in the last year. To investigate whether there was a relationship between epithelial gene signature and exacerbation frequency in this severe population we used BRB-ArrayTools (developed by Dr. Richard Simon and the BRB-ArrayTools Development Team). This did not reveal any association. The study was not powered, however, to specifically address this point and would, in all likelihood, require larger a population to adequately address this issue.

References

1. Boulet LP, FitzGerald JM, Reddel HK. The revised 2014 GINA strategy report: opportunities for change. Current opinion in pulmonary medicine. 2015;21(1):1-7.

2. Chung KF, Wenzel SE, Brozek JL, Bush A, Castro M, Sterk PJ, et al. International ERS/ATS guidelines on definition, evaluation and treatment of severe asthma. Eur Respir J. 2014;43(2):343-73.

3. Society AT. European Respiratory Society. ATS/ERS recommendations for standardized procedures for the online and offline measurement of exhaled lower respiratory nitric oxide and nasal nitric oxide, 2005. Am J Respir Crit Care Med. 2005;171(8):912-30.

4. Committee BTSBG. British Thoracic Society guidelines on diagnostic flexible bronchoscopy. Thorax. 2001;56(suppl 1):i1-i21.

5. Wu Z, Irizarry RA, Gentleman R, Martinez-Murillo F, Spencer F. A model-based background adjustment for oligonucleotide expression arrays. Journal of the American statistical Association. 2004;99(468):909-17.

6. Leek JT, Johnson WE, Parker HS, Jaffe AE, Storey JD. SVA: surrogate variable analysis. R package version. 2013;3(0).

7. Smyth GK. Limma: linear models for microarray data. Bioinformatics and computational biology solutions using R and Bioconductor: Springer; 2005. p. 397-420.

8. Benjamini Y, Hochberg Y. Controlling the false discovery rate: a practical and powerful approach to multiple testing. Journal of the Royal Statistical Society Series B (Methodological). 1995:289-300.

9. Chen J, Bardes EE, Aronow BJ, Jegga AG. ToppGene Suite for gene list enrichment analysis and candidate gene prioritization. Nucleic Acids Res. 2009;37(suppl 2):W305-W11.

10. Supek F, Bošnjak M, Škunca N, Šmuc T. REVIGO summarizes and visualizes long lists of gene ontology terms. PloS one. 2011;6(7):e21800.

11. Cho J-H, Wang K, Galas DJ. An integrative approach to inferring biologically meaningful gene modules. BMC systems biology. 2011;5(1):117.

12. Wang JZ, Du Z, Payattakool R, Philip SY, Chen C-F. A new method to measure the semantic similarity of GO terms. Bioinformatics. 2007;23(10):1274-81.

13. Yu G, Li F, Qin Y, Bo X, Wu Y, Wang S. GOSemSim: an R package for measuring semantic similarity among GO terms and gene products. Bioinformatics. 2010;26(7):976-8.

14. Bodenhofer U, Kothmeier A, Hochreiter S. APCluster: an R package for affinity propagation clustering. Bioinformatics. 2011;27(17):2463-4.

15. Jiang J, Malavia N, Suresh V, George SC. Nitric oxide gas phase release in human small airway epithelial cells. Respir Res. 2009;10(3).

16. Brindicci C, Ito K, Barnes PJ, Kharitonov SA. Differential flow analysis of exhaled nitric oxide in patients with asthma of differing severity. CHEST Journal. 2007;131(5):1353-62.

17. van Veen IH, Sterk PJ, Schot R, Gauw SA, Rabe KF, Bel EH. Alveolar nitric oxide versus measures of peripheral airway dysfunction in severe asthma. Eur Respir J. 2006;27(5):951-6.

18. Mahut B, Trinquart L, Le Bourgeois M, Becquemin MH, Beydon N, Aubourg F, et al. Multicentre trial evaluating alveolar NO fraction as a marker of asthma control and severity. Allergy. 2010;65(5):636-44.

19. Djukanović R, Homeyard S, Gratziou C, Madden J, Walls A, Montefort S, et al. The effect of treatment with oral corticosteroids on asthma symptoms and airway inflammation. Am J Respir Crit Care Med. 1997;155(3):826-32.

20. Bradding P, Feather IH, Wilson S, Holgate ST, Howarth PH. Cytokine immunoreactivity in seasonal rhinitis: regulation by a topical corticosteroid. Am J Respir Crit Care Med. 1995;151(6):1900-6.

21. Djukanović R, Wilson JW, Britten KM, Wilson SJ, Walls AF, Roche WR, et al. Effect of an inhaled corticosteroid on airway inflammation and symptoms in asthma. American Review of Respiratory Disease. 1992;145(3):669-74.
